# The Genetic and Embryo–Fetal Developmental Toxicity Profile of the Novel Transgelin Agonist Deg-AZM: Ames, Micronucleus, Chromosomal Aberration, and Rat EFD Studies

**DOI:** 10.3390/biomedicines13112600

**Published:** 2025-10-23

**Authors:** Xiaoting Gu, Ying Xu, Nannan Liu, Keran Li, Xiaoting Wang, Jia Zhang, Xiaoting Zhang, Yanjie Ding, Xiaohe Li, Honggang Zhou, Xiaoyu Ai, Cheng Yang

**Affiliations:** College of Pharmacy, State Key Laboratory of Medicinal Chemical Biology and Tianjin Key Laboratory of Molecular Drug Research, Nankai University, Tianjin 300350, China; guxiaoting@nankai.edu.cn (X.G.); xyying1688@163.com (Y.X.); 18032422509@163.com (N.L.); lkr13111337893@163.com (K.L.); 15606406360@163.com (X.W.); zhangjia2001@126.com (J.Z.); zhangxiaoting066@126.com (X.Z.); 18632287161@126.com (Y.D.); lixiaohe908@163.com (X.L.); honggang.zhou@nankai.edu.cn (H.Z.)

**Keywords:** Deg-AZM, preclinical toxicology, genotoxicity, embryo–fetal developmental toxicity, toxicokinetic, repeated toxicity

## Abstract

**Background:** Slow-transit constipation (STC) lacks durable and safe prokinetics. Deglycosylated-azithromycin (Deg-AZM), a novel small-molecule transgelin agonist that restores colonic motility in STC, has been approved for clinical trials in 2024. **Objectives:** This study aimed to assess the genetic toxicity and embryo–fetal development (EFD) toxicity of Deg-AZM through a series of standardized non-clinical safety studies. **Methods:** We conducted Ames, in vivo micronucleus, and chromosomal aberration tests to evaluate genotoxicity. Acute and 28-day repeated-dose oral toxicity studies were performed in Sprague-Dawley rats. EFD toxicity was assessed in pregnant rats administered Deg-AZM from gestation day (GD) 6 to 15. Toxicokinetic analyses were integrated into repeated-dose and EFD studies. **Results:** Deg-AZM demonstrated no mutagenic potential in the bacterial reverse-mutation assay at concentrations up to 2500 µg/plate (with metabolic activation) or 150 µg/plate (without metabolic activation). No clastogenic effects were observed in micronucleus or chromosomal aberration assays. The median lethal dose (LD_50_) exceeded 1600 mg/kg in acute oral toxicity. In the 28-day study, no adverse effects were observed at doses up to 600 mg/kg, though mild hematological and hepatic changes were noted at high doses, all of which were reversible. In the EFD study, Deg-AZM did not induce maternal toxicity, teratogenicity, or adverse fetal outcomes at doses up to 600 mg/kg. **Conclusions:** Deg-AZM demonstrates a favorable safety profile with no evidence of genetic toxicity or developmental harm at pharmacologically relevant doses, supporting its further development as a therapeutic agent for STC.

## 1. Introduction

The global incidence of constipation is steadily increasing due to factors including dietary shifts, sedentary behavior, aging demographics, and psychosocial influences [1,2]. Chronic constipation affects approximately 15% of the global population, with prevalence rates in China ranging from 7.3% to 20.39% according to epidemiologic studies [2,3]. Slow-transit constipation (STC) a common subtype, is characterized by infrequent bowel movements, straining, hard stools, abdominal distension, and significantly impaired quality of life [4]. The pathophysiology of STC involves complex and multifactorial mechanisms such as myopathic and neuropathic dysfunctions, which underlie the current lack of universally effective treatments [5]. First-line management primarily involves conservative approaches including laxatives such as bulk-forming, osmotic, and stimulant agents, dietary fiber supplementation, and lifestyle modifications [6]. Although pharmacological interventions like prokinetic agents, for example, 5-Hydroxytryptamine receptor subtype 4 (5-HT4) agonists such as mosapride and tegaserod and guanylate cyclase agonists, have been employed, their utility is often constrained by adverse effects including nausea, abdominal pain, diarrhea, cardiovascular risks, and drug resistance [7,8]. Notably, several previously approved drugs including cisapride have been withdrawn from the market owing to severe cardiac adverse events related to poor target selectivity [9]. Surgical interventions such as colectomy or ileostomy are considered only for severe cases refractory to medical treatment but carry risks of persistent constipation, diarrhea, bowel obstruction, and disease recurrence [10]. Furthermore, long-term use of certain herbal laxatives such as rhubarb and senna has been associated with colorectal melanosis and potential carcinogenic effects [11]. Given these substantial limitations and unmet clinical needs, there is an urgent demand for the development of novel, efficient, and low-toxicity pharmacological agents that specifically target the underlying colodynamic dysfunction in STC to restore normal intestinal motility and provide sustained symptomatic relief.

Our previous studies identified deglycosylated azithromycin (Deg-AZM) as a potent intestinal agonist that enhances contractility via a novel mechanism. Specifically, Deg-AZM acts as a small-molecule agonist of Transgelin (SM22α), a 22 kDa smooth muscle-specific actin-binding protein within the calponin family that is abundantly expressed in the gastrointestinal tract [12,13,14]. By upregulating Transgelin expression in intestinal smooth muscle cells, Deg-AZM facilitates the polymerization of globular actin (G-actin) into filamentous actin (F-actin), promotes the assembly of contractile stress fibers, and ultimately stimulates intestinal peristalsis [15]. As a first-in-class Transgelin agonist specifically developed for STC, Deg-AZM exhibits a well-defined mechanism, pronounced efficacy, and a favorable safety profile. Notably, no other transgelin-targeting agents are currently available for clinical treatment of constipation. Supported by these attributes, Deg-AZM has received implied approval for clinical trials in China (Acceptance No. CXHL2400005) and represents a promising therapeutic option for patients with STC.

Before first-in-human dosing, a comprehensive safety characterization covering genotoxicity, EFD and dose range toxicity is mandated by ICH M3 (R2) and ICH S5 (R3) guidelines [16,17]. Although our previous reports in Beagle dogs defined an initial no-observed-adverse-effect level and revealed reversible, dose-dependent cardiopulmonary findings at supratherapeutic exposures [18], the genetic and reproductive toxicology of Deg-AZM had not been scrutinized. The aim of this study was to conduct a comprehensive preclinical safety evaluation of Deg-AZM, incorporating a series of genotoxicity and developmental toxicity assessments. These included the Ames test to detect gene mutations, an in vitro chromosomal aberration assay to identify structural chromosomal damage, an in vivo micronucleus test to evaluate genotoxicity at the whole-animal level, and a rat EFD study to directly assess effects on maternal health and fetal development. Additionally, single-dose and 28-day repeat-dose studies in Sprague–Dawley rats were performed to characterize systemic toxicity. These studies provide a holistic preclinical safety profile essential for supporting the risk-benefit assessment and clinical development plan of Deg-AZM as a novel small-molecule agonist for STC.

## 2. Materials and Methods

### 2.1. Chemicals, Reagents and Materials

Deg-AZM was obtained from Nankai University (Tianjin, China). Dimethyl sulfoxide (DMSO) was obtained from Solarbio (Beijing, China). Mitomycin C was supplied by Shanghai Yingxin Laboratory Equipment Co., Ltd. (Shanghai, China). Cyclophosphamide was produced by Baxter Oncology GmbH (Halle, Germany). Carboxymethylcellulose sodium (CMC-Na) was purchased from Sinopharm Chemical Reagent Co., Ltd. (Shanghai, China). Deionized water employed for all procedures was obtained from Wahaha Corporation (Hangzhou, China).

### 2.2. Animals

Sprague-Dawley (SD) rats and BALB/c mice were employed across various toxicity studies. SD rats of both sexes were utilized in single-dose, reproductive, and long-term toxicity evaluations. In the single-dose and reproductive toxicity tests, rats aged 7–10 weeks were used, with body weights ranging from 216.2 to 244.4 g for males and 187.5–220.1 g for females. For the long-term toxicity study, 5–7-week-old SD rats were selected, weighing 150.7–176.0 g (males) and 128.1–173.8 g (females). Additionally, male BALB/c mice aged 7–9 weeks, with body weights between 17.21 and 19.78 g, were used in the micronucleus assay. All animals were sourced from Beijing Vital River Laboratory Animal Technology Co., Ltd. (Beijing, China) and housed under barrier conditions with a controlled ambient temperature of 20–26 °C, relative humidity of 40–70%, and a 12 h light/dark cycle. Overnight fasting was implemented prior to dosing in applicable studies. All experimental procedures were conducted in facilities accredited by the International Laboratory Animal Evaluation and Certification Management Committee and were approved by the Institutional Animal Care and Use Committee of Shandong Xibo Drug Safety Evaluation Research Center (Approval Nos: XB-IACUC-2020-0194).

### 2.3. Genetic Toxicology Studies

#### 2.3.1. Ames Test

The Ames test was conducted in accordance with the OECD Guideline 471 (Bacterial Reverse Mutation Test) [19] and in compliance with the guidelines for genotoxicity testing and data interpretation of pharmaceuticals intended for human use. Five histidine-auxotrophic Salmonella typhimurium strains, TA97a, TA98, TA100, TA102, and TA1535, were obtained from Shanghai Baolu Biological Technology Co., Ltd. (Shanghai, China). Each strain underwent genetic authentication to confirm identity and functional responsiveness. The assay was performed both with and without metabolic activation using a rat liver *S9* fraction (Moltox, batch no. 4134) induced by Aroclor 1254. Bacterial cultures were grown in nutrient broth under shaking to achieve approximately 1–2 × 10^9^ cells/mL. Deg-AZM was DMSO to prepare the test solutions with the concentration at 1.85, 5.56, 16.67, 50, and 150 μg/plate without metabolic activation, and 30.86, 92.59, 277.78, 833.33, and 2500 μg/plate with metabolic activation. Strain-specific positive controls (CTL) were employed: without *S9*, 9-aminoacridine (50 μg/plate) for TA97a; 2,7-diaminofluorene (20 μg/plate) for TA98; sodium azide (1.5 μg/plate) for TA100 and TA1535; and mitomycin C (0.5 μg/plate) for TA102. With *S9* activation, 2-aminofluorene (50 μg/plate) was used for all strains. DMSO was used as the vehicle CTL.

The pre-incubation method was applied throughout the study. Briefly, 0.1 mL of bacterial culture, 0.1 mL of test article or CTL substance, and 0.5 mL of *S9* mixture (or phosphate buffer for assays without activation) were combined and incubated at 37 °C for 20 min. Following incubation, 2 mL of top agar containing histidine-biotin was added, and the mixture was poured onto minimal glucose (GLU) agar plates. After the top agar solidified, the plates were inverted and incubated at 37 °C for 48–72 h. Revertant colonies were counted manually. Each concentration was tested in triplicate, and the entire experiment was independently repeated twice.

#### 2.3.2. In Vivo Mammalian Micronucleus Test

The in vivo mammalian micronucleus assay was performed following the OECD Guideline 474 (Mammalian Erythrocyte Micronucleus Test) [20] to evaluate the mutagenic potential of the test substance. Fifty male BALB/c mice were randomly assigned to five groups (n = 10 per group): a vehicle CTL group, three groups administered Deg-AZM at doses of 120, 300, and 750 mg/kg, and a positive CTL group receiving cyclophosphamide (50 mg/kg). All treatments were administered orally on two occasions separated by a 24 h interval. Bone marrow samples were collected from the sternum at 24 h and 48 h after the final administration, with five animals per group humanely euthanized at each time point. The samples were then prepared into suspensions, smeared, and fixed in methanol for ten minutes. After air-drying, slides were stained with Giemsa staining solution for 15 min. Under oil-immersion microscopy, polychromatic erythrocytes (PCE) were identified by their gray-blue appearance, while normochromic erythrocytes (NCE) stained pink. Characteristic micronuclei (MN) appeared as single, round, well-defined, chromophobic structures within the cytoplasm, exhibiting purplish-red to bluish-purple coloration and measuring approximately 1/20 to 1/5 of the diameter of the host erythrocyte. To ensure objectivity, a rigorous process was followed in which 4000 Giemsa-stained erythrocytes were counted per animal slide specimen through double-blind readings. The presence of MN in PCE was documented, and the micronucleus rate was expressed per thousand. Furthermore, the number of PCE among a total of 500 erythrocytes (comprising both PCE and NCE) was quantified, yielding the PCE/(PCE + NCE) ratio).

#### 2.3.3. Chromosomal Aberration Test of Chinese Hamster Lung (CHL) Cells

The chromosomal aberration assay was conducted in compliance with the OECD Guideline 473 (In Vitro Mammalian Chromosomal Aberration Test) [21]. CHL cells were seeded in 24-well plates at a density of 2 × 10^5^ cells per well and cultured for 24 h in a humidified incubator at 37 °C with 5% CO_2_. The study was conducted under two metabolic activation conditions: with *S9* mix (+*S9*) and without *S9* mix (−*S9*). Four dose groups (54.25, 108.5, 217, and 434 μg/mL) were evaluated, along with a vehicle CTL (RPMI-1640 medium supplemented with 10% fetal bovine serum) and positive CTL (20 μg/mL cyclophosphamide for +*S9*; 0.25 μg/mL mitomycin C for −*S9*). Under metabolic activation (+*S9*), cells were treated with test substances in the presence of 0.5 mL *S9* mix for 4 ± 1 h, followed by replacement with fresh medium and further incubation for 24 ± 2 h. In the absence of metabolic activation (−*S9*), cells were continuously exposed to test compounds for either 24 ± 2 h or 48 ± 4 h. All treatment groups were supplemented with 0.8 μg/mL colchicine 4 ± 1 h prior to harvest to arrest cells at metaphase. The cells were harvested by digestion with 0.25% trypsin, treated with hypotonic solution (0.075 mol/L KCl in water), fixed with fixative solution (methanol:glacial acetic acid = 3:1), dripped onto pre-cooled clean slides, air-dried, and stained with Giemsa staining solution for 25 min. Each slide specimen was observed for 300 mid-division phases under an oil microscope, and structural aberrations of chromosomes, numerical aberrations, as well as the number of aberrant cells and the type of aberrations were recorded separately.

### 2.4. Single-Dose Acute Oral Toxicity Study in Rats

The acute oral toxicity study was conducted based on the principles of OECD Guideline 425 (Up-and-Down Procedure) for dose range finding and LD_50_ estimation, utilizing a fixed-dose design for initial screening [22]. Additionally, the study design considered the general recommendations of OECD Guideline 420 (Acute Oral Toxicity–Fixed Dose Procedure) [23]. Forty healthy SD rats (20 males/20 females) were divided by sex and body weight into four groups (n = 5/sex/group): a CTL group receiving 0.5% CMC-Na and three treatment groups administered Deg-AZM at doses of 800, 1200, and 1600 mg/kg (suspended in 0.5% CMC-Na). Following a single oral administration, all animals were closely monitored for abnormal signs and mortality at designated time points on the day of dosing and twice daily (morning and afternoon) for 14 consecutive days. Mortality, toxic responses, and body weight were recorded throughout the study. Body weights were measured prior to dosing and on days 2, 3, 5, 7, 10, and 14 post-administration, while 24 h food consumption was assessed twice per week. All deceased animals, as well as surviving animals at the end of the study, underwent gross necropsy, and any organs with macroscopic abnormalities were subjected to histopathological examination.

### 2.5. 28-Day Repeated-Dose Oral Toxicity Study in Rats

This 28-day repeated-dose oral toxicity study was conducted in accordance with the principles of OECD Guideline 407 [24]. A total of 180 healthy SD rats (90 males/90 females) were stratified by sex and body weight and allocated into six experimental groups (n = 30/sex/group), consisting of a CTL group and five treatment groups administered Deg-AZM at doses of 10, 25, 50, 150, and 600 mg/kg (suspended in 0.5% CMC-Na). The CTL group received the 0.5% CMC-Na vehicle solution, while treated animals were administered Deg-AZM at the corresponding doses via oral gavage once daily in the morning for 28 consecutive days at a dosing volume of 10 mL/kg, followed by a 28-day recovery period.

To characterize systemic exposure, a separate cohort of eighty-four healthy SD rats (42 males/42 females) was established as satellite groups exclusively for toxicokinetic analysis. These animals were distinct from those in the main study groups and were not used for any other endpoints. They were randomly allocated into CTL group (n = 4) and different dose groups of Deg-AZM (10, 25, 50, 150, and 600 mg/kg groups, n = 16 per group) based on gender and body weight, with dosing regimens identical to those in the study groups.

#### 2.5.1. Toxicological Observations

During the quarantine and acclimatization periods of the study, SD rats were monitored once daily for clinical signs. Following the initiation of dosing, all animals were closely monitored for abnormal clinical findings and mortality at specified time points on dosing days (pre-dose, within 60 min post dose, and in the afternoon). On non-dosing days, monitoring was performed twice daily (morning and evening). Body weights were recorded weekly throughout acclimation and twice weekly during the dosing phase for all surviving animals. Food consumption was assessed daily by providing each rat with a pre-weighed quantity of food in the afternoon; remaining food was weighed the following afternoon. Ophthalmic examinations were performed using a YZ25B binocular indirect ophthalmoscope (Suzhou 66 Vision Tech Co., Ltd., Suzhou, China) before dosing, at the end of the treatment period, and at the end of the recovery period.

#### 2.5.2. Hematological Examination and Coagulation Tests

Hematological and coagulation analyses were performed on all animals scheduled for necropsy following the completion of drug administration. For hematological examination, approximately 0.5 mL of blood was collected from the inferior vena cava (IVC) of each SD rat into EDTA-anticoagulated tubes. Samples were analyzed using an ADVIA 2120 hematology analyzer (Siemens Healthineers, Shanghai, China). Parameters assessed included red blood cell count (RBC), hematocrit (HCT), hemoglobin concentration (HGB), mean corpuscular volume (MCV), mean corpuscular hemoglobin (MCH), mean corpuscular hemoglobin concentration (MCHC), red cell distribution width (RDW), white blood cell count (WBC), platelet count (PLT), platelet distribution width (PDW), leukocyte differential counts and percentages (LYM%, NEU%, BASO%, EOS%, MONO%, and absolute counts LYM, NEU, BASO, EOS, MONO), and reticulocyte percentage (RETIC%).

Coagulation testing was conducted using approximately 1 mL of IVC blood collected into sodium citrate anticoagulant tubes. Plasma was separated by centrifugation at 3000 rpm for 10 min and analyzed on an ACL TOP 300 CTS automated coagulation analyzer (Werfen, Bedford, MA, USA). Measured parameters comprised prothrombin time (PT), activated partial thromboplastin time (APTT), thrombin time (TT), and fibrinogen (FIB).

#### 2.5.3. Serum Biochemical Analysis

Serum biochemical parameters were assessed in all animals scheduled for necropsy upon completion of drug administration. Evaluated indices included aspartate aminotransferase (AST), alanine aminotransferase (ALT), alkaline phosphatase (ALP), total bilirubin (TBIL), total protein (TP), albumin (ALB), globulin (GLO), albumin-to-globulin ratio (A/G), blood urea nitrogen (BUN), creatinine (CREA), total cholesterol (CHOL), triglycerides (TGL), GLU, creatine kinase (CK), gamma-glutamyl transferase (GGT), and electrolytes (potassium [K], sodium [Na], chloride [Cl], calcium [Ca], phosphorus [PHOS]). Approximately 3 mL of blood was collected from limb veins into anticoagulant-free plastic tubes. The samples were allowed to clot at room temperature and then centrifuged at 3000 rpm for 10 min to obtain serum, which was subsequently analyzed using the Dimension RxL Max fully automated biochemistry analyzer (Siemens Healthineers, Shanghai, China).

#### 2.5.4. Toxicokinetic Analysis in 28-Day Repeated-Dose Oral Toxicity Study

Toxicokinetic blood sampling was performed in satellite group rats following the first dose and the last dose. Blood samples were collected pre-dose and at 2 min (±5 s), 10 min (±5 s), 30 min (±20 s), 1 h (±2 min), 2 h (±2 min), 4 h (±2 min), 6 h (±2 min), 10 h (±2 min), and 24 h (±5 min) post-dose. For the vehicle CTL satellite group, blood was collected only pre-dose and at 1 h (±2 min) post-dose. Approximately 0.2 mL of venous blood was collected from the rat jugular venous sinus into heparinized tubes. The samples were centrifuged at 12,000 rpm for 3 min to separate the plasma. The harvested plasma was transferred into microtubes and temporarily stored at −20 °C during the sampling process prior to analysis for determining the concentration of drug.

Non-compartmental pharmacokinetic analysis was performed on individual concentration–time profiles using DAS 3.3 software (Chinese Pharmacological Association, Beijing, China). Key parameters assessed included T_max_ (time to maximum concentration), C_max_ (peak concentration), AUC (area under the concentration–time curve), and T_1/2_ (terminal elimination half-life).

#### 2.5.5. Pathological Examination

Pathological examinations were conducted on twenty sex-balanced animals per main study group at the termination of the treatment period and on ten sex-balanced animals per main study group after the recovery phase. Satellite group animals were excluded from these analyses. Under anesthesia induced by intraperitoneal injection of tiletamine-zolazepam hydrochloride (30 mg/kg, 0.6 mL/kg of a 50 mg/mL solution), the animals were euthanized via exsanguination and subjected to thorough gross examination. Absolute weights of the brain, lungs, thymus, heart, liver (including gallbladder), spleen, kidneys, adrenals, testes, epididymides, ovaries, uterus, and thyroid (including parathyroid) were recorded for the calculation of organ-to-body weight ratios (g/kg). All weighed organs, together with the following tissues, were collected for histopathological processing: spinal cord (cervical, thoracic, lumbar segments), pituitary gland, trachea, esophagus, submandibular glands, stomach, duodenum, jejunum, ileum, cecum, colon, rectum, pancreas, aorta, eyes, oviducts, prostate, abdominal skin, mammary glands (in females), vagina, sciatic nerves, bladder, optic nerves, sternum (with marrow), femur (with marrow), skeletal muscle, submandibular and mesenteric lymph nodes, tongue, and any tissues with macroscopic observations.

Eyes and male reproductive organs were fixed in Davidson’s solution for 24 h before transfer to 10% neutral buffered formalin (NBF); all other tissues were fixed directly in 10% NBF. All specimens were subsequently embedded in paraffin, sectioned, and stained with hematoxylin and eosin (H&E) for microscopic evaluation.

### 2.6. EFD Toxicity Study in SD Rats

The embryo–fetal developmental toxicity study was performed based on OECD Guideline 414 (Prenatal Developmental Toxicity Study) [25]. A total of 160 female and 80 male SD rats were used in this study. Males were used solely for mating and were not assigned to experimental groups. They were co-housed with females at a 1:1 ratio. Vaginal plugs were checked the following morning; females without visible plugs were further examined via vaginal smear for the presence of sperm. The observation of a vaginal plug or sperm was defined as successful mating, and that day was designated as GD 0.

For the main trial, 105 mated females were randomly assigned by body weight to a vehicle CTL group (n = 23) and Deg-AZM treatment groups (50, 150, and 600 mg/kg groups; n = 23, 24, and 24, respectively). An additional toxicokinetic evaluation involved 55 healthy female rats, which were also randomized by body weight into a CTL group (n = 12) and Deg-AZM dose groups (50, 150, and 600 mg/kg; n = 12 per group). All animals were administered the test articles via oral gavage: the CTL group received 0.5% CMC-Na solution, while the Deg-AZM groups received respective doses once daily from GD 6 to 15 at a volume of 10 mL/kg/day, followed by a 28-day recovery period.

For toxicokinetic analysis, six pregnant rats per batch were used: the first batch received a single administration on GD 6, and the second batch received repeated daily administrations from GD 6 to 15.

#### 2.6.1. Toxicological Observations

Throughout the quarantine and acclimation phases, clinical observations of female rats were conducted once per day. Following the commencement of compound administration, animals were evaluated three times per day on dosing days (pre-dose in the morning, 20–60 min post dose, and in the afternoon) and twice daily on non-dosing days (both morning and afternoon). Assessed endpoints included incidence of pseudopregnancy, conception, mortality, preterm delivery, and abortion.

Body weights were recorded for all surviving animals on GD 0, 3, 6, 9, 12, 15, 18, and 20. Food consumption was measured for all surviving main trial animals on the mornings of GD 0, 3, 6, 9, 12, 15, 18, and 20. A predetermined quantity of food was provided daily at consistent times for ad libitum intake, with residual feed weighed and recorded the subsequent morning.

#### 2.6.2. Pregnant Rat Necropsy and Fetal Examination

Dams in the main study groups were euthanized via CO_2_ asphyxiation on GD 20 and subjected to necropsy. A comprehensive macroscopic examination was performed to evaluate external features and the gross morphology of all visceral organs and tissues. The brain was excised and its wet weight recorded. Following the collection of live offspring, a detailed external examination was conducted to assess their appearance. Approximately half of the fetuses from each litter were designated for skeletal examination. These were eviscerated, and subcutaneous fat and skin were removed, followed by fixation in 95% ethanol for two weeks. After secondary fixation, the specimens were transferred to a staining solution and immersed for approximately two days. Upon completion of staining, the solution was replaced with a clearing/bleaching agent for about two additional days. The cleared specimens were stored in 70% glycerol until detailed examination of the skeletal structures was performed using a stereomicroscope with continuous zoom capability. The remaining half of the fetuses were fixed in 10% neutral buffered formalin for a minimum of five weeks. These specimens were then evaluated for visceral abnormalities using free-hand sectioning techniques.

Dams in the satellite group were similarly euthanized by CO_2_ asphyxiation on GD 20 and underwent gross necropsy to confirm pregnancy status.

#### 2.6.3. Toxicokinetic Analysis in Pregnant Rats

Toxicokinetic blood sampling was performed in satellite group dams following compound administration on Gestation Day GD 6 (first batch) and GD 15 (second batch). The methodology for sample processing, and analysis was identical to that described in detail in Section 2.5.4. Animals in the vehicle CTL satellite group were bled only once at 10 min (±5 s) post-dose.

### 2.7. Data Statistics Method

Data were presented as mean ± standard deviation (SD) and were graphically visualized using GraphPad Prism 10.1 (GraphPad, San Diego, CA, USA). For the Ames test, in vivo micronucleus test, acute oral toxicity study, 28-day repeated-dose study, and EFD toxicity study, statistical comparisons between each dose group and the vehicle control were conducted using *t*-tests or, where applicable, a decision tree approach: Bartlett’s test was first used to assess homogeneity of variance. If homogeneous (*p* > 0.05), one-way ANOVA was applied; if significant (*p* ≤ 0.05), it was followed by Dunnett’s parametric test for multiple comparisons. For non-homogeneous data (*p* ≤ 0.05), the Kruskal–Wallis test was used, with Dunnett’s non-parametric test applied when results were significant (*p* ≤ 0.05). For the EFD study, the litter was considered the experimental and statistical unit for all fetal abnormality assessments. Categorical data (e.g., mortality, preterm rate, abortion rate, pregnancy rate) were analyzed using Fisher’s exact test (one-tailed), and sex ratio was compared using Pearson’s chi-square test. For the chromosomal aberration test, biological relevance was prioritized, with statistical analysis serving a supportive role. Toxicokinetic parameters (AUC_0–t_ and C_max_) were compared using SPSS Statistics 29.

## 3. Results

### 3.1. Genetic Toxicology

#### 3.1.1. Ames Test

To evaluate the potential mutagenicity of Deg-AZM, an Ames test was performed using histidine-deficient strains of Salmonella typhimurium (TA97a, TA98, TA100, TA102, and TA1535) both with and without metabolic activation by *S9*. As summarized in Table 1, the number of revertant colonies in all vehicle CTL groups fell within established historical control ranges, whereas a significant increase (*p* ≤ 0.01) was observed in all positive CTL groups, confirming the adequacy of the test conditions.

In two independent trials, Deg-AZM, at doses of up to 150 μg/plate (without activation) and 2500 μg/plate (with activation), did not induce a statistically significant or reproducible increase in revertant colonies in any of the tested strains (TA97a, TA98, TA100, TA102, TA1535). No dose-dependent increasing trend was observed. It was worth noting that at doses of 150 μg/plate (without activation) and 2500 μg/plate (with activation), a significant reduction (*p* ≤ 0.05) in revertant colonies was observed in strains TA97a and TA100. This phenomenon was attributed to bacterial toxicity rather than mutagenic activity. Under the conditions of this test, the Ames test results for Deg-AZM were negative across all tested strains, indicating no evidence of mutagenicity in this assay system.

#### 3.1.2. In Vivo Mammalian Micronucleus Analysis

To investigate the potential genotoxicity of Deg-AZM, its ability to induce micronucleus formation in PCEs was evaluated in male BALB/c mice following a single oral administration at doses of 120, 300, and 750 mg/kg. As summarized in Table 2, bone marrow samples were collected at 24 h and 48 h after administration. A total of 4000 PCE per animal were examined to determine the frequency of micronucleated cells and the PCE/(PCE + NCE) ratio was calculated to assess bone marrow toxicity. The positive CTL group showed significantly elevated micronucleus frequencies of (15.00 ± 2.59) ‰ and (13.05 ± 2.88) ‰ at 24 h and 48 h, respectively, along with a markedly reduced PCE ratio (*p* ≤ 0.01), confirming the sensitivity and validity of the assay. The Deg-AZM-treated groups (120, 300, and 750 mg/kg) showed micronucleus frequencies of (0.95 ± 0.72)‰, (0.85 ± 0.70)‰, and (1.25 ± 0.40) ‰ at 24 h, and (0.90 ± 0.63) ‰, (0.65 ± 0.38) ‰, and (1.00 ± 0.40) ‰ at 48 h. Compared with the vehicle CTL group (24 h: 0.90 ± 0.58 ‰; 48 h: 0.80 ± 0.48 ‰), none of the Deg-AZM-treated groups showed statistically significant differences in micronucleus frequency (*p* > 0.05). Additionally, no significant differences were observed in the PCE/(PCE + NCE) ratio between any Deg-AZM-treated group and the vehicle CTL group, indicating that Deg-AZM did not suppress bone marrow hematopoiesis. Therefore, under the experimental conditions, Deg-AZM did not induce micronucleus formation in bone marrow PCE of BALB/c mice within the dose range of 120–750 mg/kg, confirming that this compound exhibits no genotoxicity at these doses.

#### 3.1.3. Chromosomal Aberration Test of CHL Cells

To assess the potential clastogenic effects of Deg-AZM, an in vitro mammalian chromosomal aberration test was performed using CHL cells. As summarized in Table 3, microscopic evaluation revealed no precipitation of the test article at any concentration, and the cells maintained normal morphology and confluence with no evidence of overt cytotoxicity, consistent with parallel cytotoxicity assays. Under both non-activation (−*S9*) and metabolic activation (+*S9*) conditions, the positive controls mitomycin C and cyclophosphamide induced significant chromosomal aberration rates (12.3–14.3% and 11.7%, respectively), which were markedly higher (*p* ≤ 0.01) than those in the vehicle CTL group, confirming the sensitivity and reliability of the test system. Deg-AZM, at concentrations ranging from 54.25 to 434 μg/mL, did not produce a statistically significant increase in chromosomal aberration rates at any sampling time (4 h, 24 h, or 48 h), with all values remaining at or below 1.0%. No dose-dependent response was observed, and the frequency of polyploid cells did not differ notably from that of the vehicle CTL control. Under the experimental conditions applied, Deg-AZM was concluded to be non-clastogenic in CHL cells.

### 3.2. Single-Dose Acute Oral Toxicity Study in Rat

**Figure 1:**
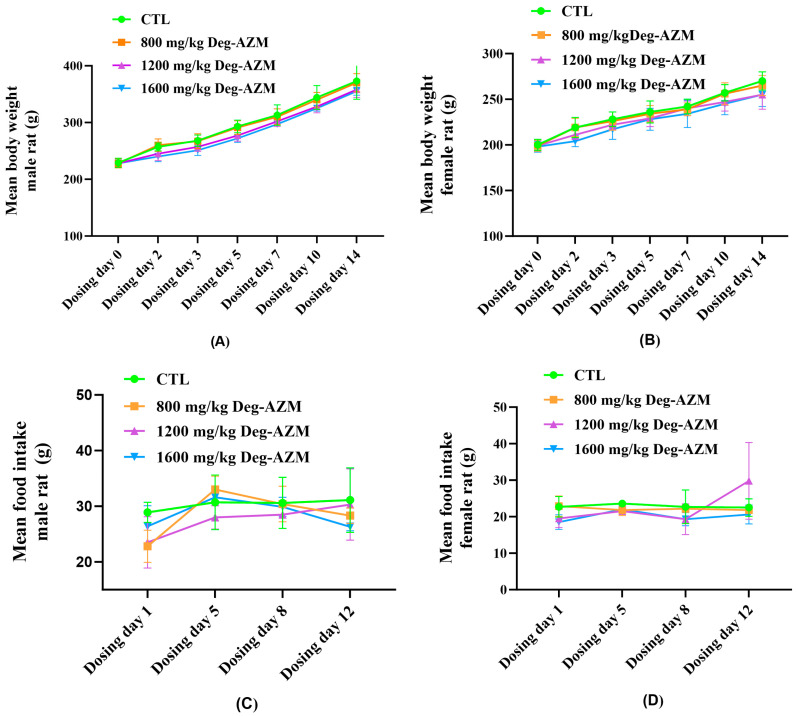
Body weight in male (**A**) and female (**B**) rats, food intake in male (**C**) and female (**D**) rats following oral administration of Deg-AZM. Data are expressed as means ± SD (n = 5).

To preliminarily characterize the acute toxicity and dose–response relationship of the test compound, rats were administered Deg-AZM orally at doses of 800, 1200, and 1600 mg/kg and observed over a 14-day period. No mortality occurred during the study. No treatment-related abnormalities were observed in mental status, physical condition, motor activity, or oral/nasal secretions in any group compared to CTL group. The results of the effects of Deg-AZM on body weight and food consumption in rats following administration are shown in Figure 1. No significant effect on body weight gain in rats was observed in all dose groups after administration of Deg-AZM. No statistically significant differences in food consumption were detected between treated and CTL groups throughout the study. Upon completion of the observation period, a detailed gross necropsy was performed on all animals. No mortality, treatment-related clinical signs, or macroscopic pathological changes were observed during the study. Based on the absence of these findings, Deg-AZM did not induce evident acute toxic effects at the doses tested under the conditions of this study. The LD_50_ for a single oral administration of Deg-AZM suspended in 0.5% CMC-Na was concluded to be greater than 1600 mg/kg. It is important to note that a more sensitive assessment of potential target organ toxicity was provided by the histopathological evaluation in the 28-day repeated-dose study.

### 3.3. 28-Day Repeated-Dose Oral Toxicity Study in Rats

#### 3.3.1. Toxicological Observations

**Figure 2:**
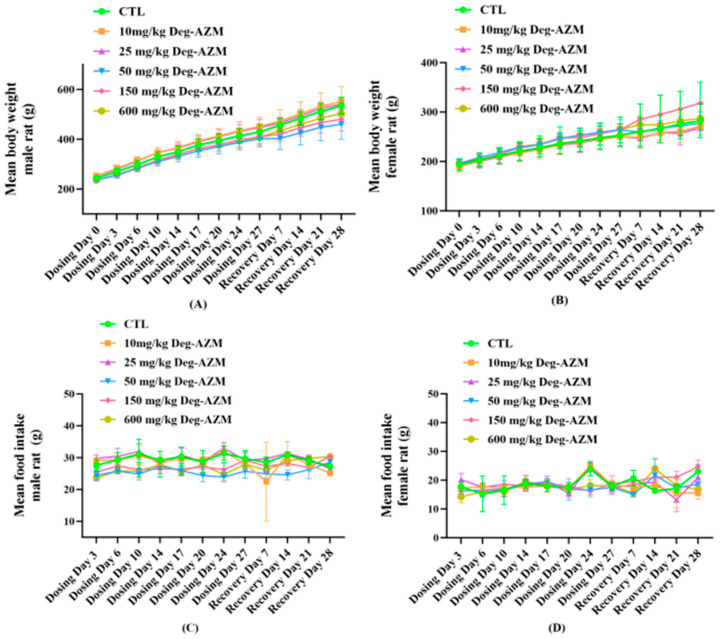
Body weight in male (**A**) and female (**B**) rats, food intake in male (**C**) and female (**D**) rats following 28-day repeated-dose oral administration of Deg-AZM. Data are expressed as means ± SD, n = 15 in (**A**,**B**); n = 6 in (**C**,**D**).

During the study period, rats in the CTL group exhibited normal general conditions without observable abnormalities, and no mortality or moribundity was observed in any group. The rats in 10, 25 and 150 mg/kg Deg-AZM group maintained good health status with no clinical signs. At the 4th week of drug administration, loose stools were occasionally observed in the 50 mg/kg group (1/30), salivation was observed sporadically in animals administered 600 mg/kg (2/30). The Deg-AZM and CTL groups showed similar growth trajectories in body weight measurements, with no abnormalities noted in the comparative evaluation (Figure 2A,B). Complete feed consumption was maintained across all treatment groups without exception. On day 6 of dosing, male rats in the 50 and 600 mg/kg group exhibited slightly decreased food intake. With continued administration, no abnormal changes in food consumption were observed in any treatment groups. Thus, the fluctuations in food intake during the initial phase of dosing were considered transient and without toxicological significance. At all other time points, the food consumption of the treated animals was consistent with that of the vehicle CTL group (Figure 2C,D).

#### 3.3.2. Hematological Examination and Coagulation Tests

The results of hematological and coagulation tests in SD rats following repeated oral administration of Deg-AZM were shown in Table 4. Following 28 days of repeated administration and comparison with the CTL group, no abnormalities were observed in hematological or coagulation parameters in the 25 mg/kg group, male animals in the 10 mg/kg group showed a decrease in (MONO count, which was not considered clinically significant. No other remarkable abnormalities were noted. In the 50, 150 and 600 mg/kg groups, male animals exhibited a significant reduction in eosinophil (EOS) count after 27 days of continuous dosing, while all other parameters remained within normal limits. In the 150 and 600 mg/kg group, PLT counts were decreased by approximately 9.3% and 8.0%, respectively, compared to the vehicle CTL group, and MCV was slightly increased. By the end of the recovery period, all parameters in each group had returned to normal ranges. These observed changes in hematological parameters, including decreased MONO, significantly reduced EOS, mildly lowered PLT, and slightly elevated MCV, were mild and transient, with complete recovery during the recovery period. Furthermore, these changes were not accompanied by any other related toxicological effects, and the magnitude of change (e.g., the decrease in PLT was well below the widely accepted threshold of biological significance, typically 25–30%) was insufficient to be considered adverse. Therefore, these alterations are collectively regarded as adaptive physiological responses of the animals to the drug administration and are not considered to have toxicological significance. These findings collectively support the absence of clinically meaningful hematological or coagulation alterations following Deg-AZM administration at the studied dose levels.

#### 3.3.3. Serum Biochemical Analysis

Serum biochemical parameters were evaluated in SD rats following 28-day repeated oral administration of Deg-AZM, as summarized in Table 5. Compared with the control group, several isolated changes were noted in male rats. Increases in CREA and GLU were observed in the 10 mg/kg group, the 25 mg/kg group exhibited a decrease in ALP and an increase in GLU, no changes were detected in the 50 mg/kg group, while reductions in ALT were seen in the 150 and 600 mg/kg groups. All other parameters remained within normal physiological ranges. The elevated CREA in the 10 mg/kg male animals was not observed in higher dose groups and was not supported by any treatment-related renal histopathological findings, indicating that this change lacks toxicological relevance. The decreases in ALP, ALT, and BUN observed during the study were considered devoid of clinical significance. The increases in GLU noted in the 10 and 25 mg/kg male groups were primarily attributable to statistical differences resulting from lower concurrent vehicle CTL values, with no biological implications. In conclusion, no Deg-AZM-related alterations were observed in serum biochemical parameters across all dose groups.

#### 3.3.4. Toxicokinetic Analysis in 28-Day Repeated-Dose Oral Toxicity Study

Plasma concentrations of Deg-AZM were quantified using LC-MS/MS to characterize exposure–time profiles following oral administration in SD rats. Rats received repeated doses of 10, 25, 50, 150, and 600 mg/kg, and key pharmacokinetic parameters were summarized in Table 6. Dose-proportional increases in AUC_0–t_ and C_max_ were observed across the five dose groups (dosing ratio 1:2.5:5:15:60). After the first administration, male rats exhibited AUC_0–t_ and C_max_ ratios of 1:4.7:10.8:50.7:344.5 and 1:4.2:12.7:36.7:122.7, respectively, while females showed ratios of 1:3.1:6.68:32.4:53.7 for AUC_0–t_ and 1:2.5:5.2:21.0:21.7 for C_max_. At the end of the dosing period, the ratios were 1:4.1:10.3:24.7:157.5 (AUC_0–t_) and 1:4.4:5.6:11.2:43.1 (C_max_) in males, and 1:2.8:8.13:19.5:126.2 (AUC_0–t_) and 1:2.8:4.5:6.25:32.4 (C_max_) in females. Accumulation factors, calculated as the ratio of terminal to initial AUC_0–t_ after 28 days of dosing, were 1.2, 1.0, 1.2, 0.6, and 1.0 for the 10, 25, 50, 150, and 600 mg/kg dose groups, respectively, indicating minimal accumulation of Deg-AZM.

#### 3.3.5. Pathological Examination

The organ-to-body weight ratios in rats following repeated oral administration of Deg-AZM are summarized in Table 7. At the end of the dosing period, compared with the CTL group, male animals in the 10 mg/kg group showed a decrease in kidney-to-body weight ratio, while the liver coefficient was significantly increased in the 600 mg/kg group; however, no significant changes in organ coefficients (the ratio of organs to body weight) were observed in the 25, 50, and 150 mg/kg groups. The alteration in the kidney coefficient in the 10 mg/kg males lacked a dose–response relationship and was therefore considered unrelated to the test article. The increased liver coefficient in the 600 mg/kg group was accompanied by histopathological findings of centrilobular hypertrophy, which was considered a test article-related change. This change returned to a level comparable to the vehicle control group by the end of the recovery period.

No remarkable gross findings were observed in the 10 and 50 mg/kg groups. In the 25 mg/kg group, one animal had scattered renal hemorrhagic spots, confirmed as chronic progressive nephropathy (CPN) histologically; another showed reduced testicular and epididymal size, with histopathology indicating testicular atrophy and decreased sperm in the epididymis, both considered spontaneous. In the 150 mg/kg group, one animal had a nodule in the ileum, diagnosed as diverticulum formation; another exhibited thymic hemorrhage with no other significant histopathological changes. The ileal lesion showed no time- or dose-dependence and was spontaneous; thymic hemorrhage was likely procedure-related. No notable gross pathology was observed in any group at the end of the recovery period.

**Figure 3:**
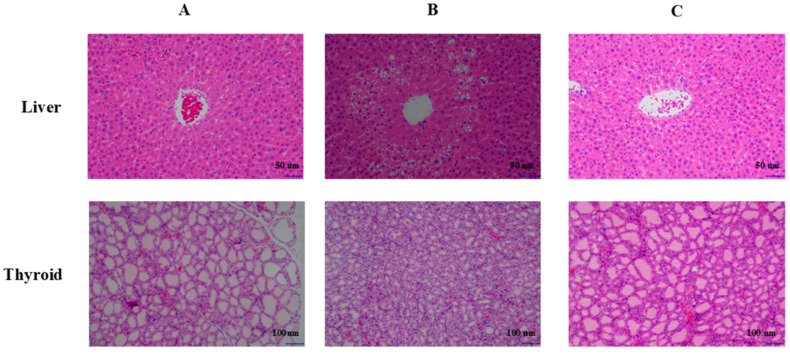
Representative histopathological photomicrographs in rats following 28-day repeated-dose oral administration of Deg-AZM. (**A**) Control group. (**B**) 600 mg/kg Deg-AZM group. (**C**) 600 mg/kg Deg-AZM group after the recovery period.

Histopathological examination demonstrated an absence of test article-related findings across all tissues in the 10 and 25 mg/kg groups upon completion of both the dosing and recovery phases. At higher dose levels of 50, 150, and 600 mg/kg, thyroid follicular epithelial cells exhibited varying degrees of diffuse hyperplasia, while hepatic alterations characterized by centrilobular hypertrophy and midzonal fatty change were specifically noted in the 150 and 600 mg/kg cohorts. Representative photomicrographs of these findings were presented in Figure 3. Importantly, all documented histopathological alterations demonstrated complete reversibility by the conclusion of the recovery period, supporting their interpretation as adaptive physiological reactions to metabolic induction rather than manifestations of compound-specific toxicity.

### 3.4. EFD Toxicity Study in Rats

#### 3.4.1. General Observations on Pregnant Rats

**Figure 4:**
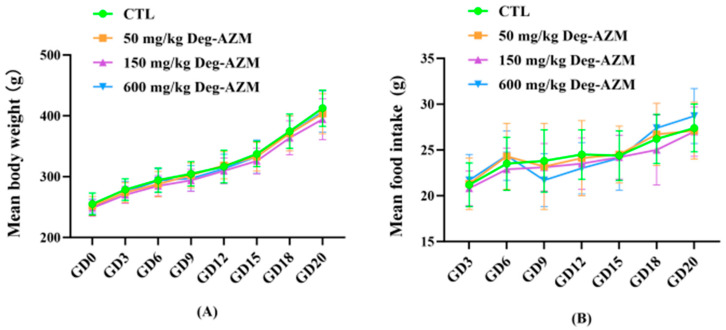
Body weight (**A**) and food intake (**B**) in pregnant rats following oral administration of Deg-AZM. Data are expressed as means ± SD (n = 21).

Throughout the study period, no test article-related clinical signs or mortality were observed in pregnant rats across all dose groups. All animals exhibited a steady increase in body weight without notable signs of anorexia. Intergroup comparisons showed no statistically significant differences in body weight change (Figure 4A) or food consumption (Figure 4B).

Pregnancy and delivery outcomes were summarized in Table 8, no statistically significant differences were observed in the frequency or incidence of pseudopregnancy, conception, mortality, preterm delivery, or abortion in the 50, 150, and 600 mg/kg Deg-AZM groups compared with the CTL group.

#### 3.4.2. Embryo–Fetal Developmental Toxicity

No statistically significant differences were observed between the CTL group and any Deg-AZM-treated group (50, 150, and 600 mg/kg) with respect to litter weight, ovarian wet weight, absolute or relative organ weights, number of corpora lutea, or implantation sites. Placental weight, fetal body weight, and crown-rump length were also comparable across all groups, as was the fetal sex ratio (Table S1). These results indicated that Deg-AZM did not induce any significant embryo–fetal developmental toxicity at the doses tested.

#### 3.4.3. Teratogenic Evaluation

Skeletal evaluations were conducted on fetuses from the CTL group and the 600 mg/kg Deg-AZM group, and the results are summarized in Table S2. No significant differences in the number of ossification sites for metacarpals, metatarsals, sternebrae, or sacrocaudal vertebrae was observed. Common skeletal variations (e.g., incomplete ossification of parietal, interparietal, occipital, or hyoid bones; dumbbell-shaped vertebral ossification; sternebral anomalies) were observed in both CTL and high-dose groups. The overall incidence of skeletal variations was similar between the vehicle CTL (83.1 ± 18.1%; 131/161 fetuses) and the 600 mg/kg group (79.9 ± 18.6%; 124/157 fetuses). No malformations were observed in any group.

Visceral examination was performed on fetuses from the CTL group and the 600 mg/kg Deg-AZM group, and the results are summarized in Table S3, revealed variations such as umbilical artery displacement, thymic lobes, dilated renal pelvis (unilateral or bilateral), and ureter dilation in both groups. The incidence of visceral variations, calculated as the mean percentage per litter, did not differ significantly between the CTL (18.6 ± 16.7%; 25/146 fetuses) and the 600 mg/kg group (20.3 ± 19.4%; 28/146 fetuses). No visceral malformations were observed. In summary, Deg-AZM administration did not result in any treatment-related teratogenic effects at doses up to 600 mg/kg.

#### 3.4.4. Toxicokinetic Analysis in Pregnant Rats

After repeated oral administration of Deg-AZM in pregnant rats (50, 150 and 600 mg/kg), the key pharmacokinetic parameters were summarized in Table 9. The three dose groups (50, 150, and 600 mg/kg) exhibited a dose ratio of 1:3:12. On GD 6, the ratios of AUC_0–t_ and C_max_ across dose groups were 1:4.0:17.0 and 1:3.4:7.9, respectively. On GD 15, the corresponding ratios were 1:4.2:18.5 and 1:3.2:10.8. The accumulation factors (calculated as AUC_0–t_ on GD 15/AUC_0–t_ on GD 6) for each dose group were 0.5, 0.6, and 0.6, respectively. Accumulation factors of 0.5–0.6 indicate lower systemic exposure on GD 15 than on GD 6, most likely due to pregnancy-induced increases in hepatic clearance. Thus, no drug accumulation was evident.

## 4. Discussion

The comprehensive preclinical safety assessment of Deg-AZM conducted in this study demonstrated a favorable genotoxic and developmental toxicity profile, supporting its potential as a novel therapeutic agent for constipation. Our results indicate that Deg-AZM is non-mutagenic, non-clastogenic, and well tolerated in both acute and subchronic dosing regimens, with no adverse effects on EFD up to the highest dose tested (600 mg/kg).

The absence of mutagenicity in the Ames test across five Salmonella typhimurium strains, both with and without metabolic activation, suggested that Deg-AZM does not interact directly with bacterial DNA or produce mutagenic metabolites. It was particularly noteworthy given that some macrolide antibiotics, such as erythromycin and clarithromycin, have been associated with weak mutagenic responses in certain in vitro systems [26]. The reduction in revertant colonies at high concentrations was attributed to cytotoxic effects rather than mutagenicity, a phenomenon also observed with other non-genotoxic compounds [27]. Similarly, the in vivo micronucleus test and chromosomal aberration assay in CHL cells revealed no significant clastogenic or aneugenic activity, further corroborating the genetic safety of Deg-AZM.

In the 28-day repeated-dose study, Deg-AZM was well tolerated with no overt signs of toxicity. The mild hematological and biochemical alterations observed at high doses (e.g., slight reductions in platelets and eosinophils, transient changes in liver enzymes) were reversible and consistent with adaptive physiological responses rather than frank toxicity. Such changes were often seen with compounds that induce hepatic metabolic enzymes. The lack of histopathological correlates except for mild centrilobular hypertrophy—a common adaptive response to enzyme induction—supports this interpretation [28]. These findings are aligned with those from previous studies in Beagle dogs, which also identified a high no-observed-adverse-effect level and reversible cardiopulmonary effects only at supratherapeutic exposures [18].

The EFD study revealed no evidence of teratogenicity, embryolethality, or maternal toxicity at doses up to 600 mg/kg. The incidences of skeletal and visceral variations were comparable between control and treatment groups, falling within historical control ranges for SD rats [29]. This robust safety profile is particularly noteworthy given that some gastrointestinal prokinetic agents have demonstrated developmental concerns in preclinical models. For example, the non-selective 5-HT4 agonist cisapride caused severe embryonic bradycardia and limb-reduction defects in rats at 75–200 mg/kg, an effect linked to hERG-channel inhibition and embryonic hypoxia [30]. These findings highlighted how promiscuous pharmacological activity can lead to direct embryonic toxicity through mechanisms beyond primary target engagement. The favorable developmental safety profile of Deg-AZM may be attributed to its highly targeted action on Transgelin—a smooth-muscle-specific protein—thereby minimizing off-target activity in embryonic tissues [15]. This target specificity represents a significant advantage over broader-spectrum prokinetic agents that interact with multiple receptor systems potentially involved in developmental processes. The absence of developmental toxicity at doses substantially exceeding anticipated therapeutic exposure provides a wide safety margin for clinical development, addressing a critical regulatory requirement for new chemical entities targeting gastrointestinal disorders.

## 5. Conclusions

In summary, Deg-AZM demonstrated no evidence of genotoxic or developmental toxicities across a comprehensive preclinical assessment. The compound was non-mutagenic, non-clastogenic, and well tolerated in acute and subchronic studies, with an LD_50_ > 1600 mg/kg. No maternal or fetal adverse effects were observed at doses up to 600 mg/kg. These results support the safety of Deg-AZM and warrant its continued clinical development for STC.

## Figures and Tables

**Table 1:** Ames test of Deg-AZM in the absence and presence of S9 metabolic activation.

	Group	TA97a	TA98	TA100	TA102	TA1535
−*S9*	vehicle CTL	82 ± 4	36 ± 6	111 ± 15	203 ± 10	16 ± 3
	1.85 μg/plate	83 ± 12	35 ± 7	106 ± 12	196 ± 14	15 ± 1
	5.56 μg/plate	89 ± 4	35 ± 8	102 ± 10	196 ± 13	15 ± 3
	16.67 μg/plate	90 ± 7	32 ± 6	104 ± 8	216 ± 11	14 ± 2
	50 μg/plate	86 ± 6	37 ± 5	116 ± 6	208 ± 24	14 ± 3
	150 μg/plate	53 ± 4 **	33 ± 4	54 ± 16 *	216 ± 9	15 ± 3
	positive CTL	883 ± 60 **	423 ± 23 **	759 ± 23 **	1033 ± 40 **	324 ± 21 **
+*S9*	vehicle CTL	103 ± 12	38 ± 1	106 ± 2	225 ± 7	16 ± 3
	30.86 μg/plate	104 ± 5	37 ± 5	100 ± 10	237 ± 11	19 ± 3
	92.59 μg/plate	105 ± 8	31 ± 6	114 ± 7	230 ± 28	16 ± 2
	277.78 μg/plate	105 ± 5	37 ± 6	106 ± 11	229 ± 15	17 ± 3
	833.33 μg/plate	105 ± 16	37 ± 8	113 ± 9	226 ± 13	22 ± 5
	2500 μg/plate	61 ± 7 **	36 ± 5	57 ± 11 *	207 ± 13	18 ± 4
	positive CTL	1065 ± 40 **	487 ± 15 **	873 ± 30 **	1191 ± 49 **	335 ± 19 **

Data are expressed as mean ± SD (n = 3). * *p* < 0.05, ** *p* < 0.01, vs. vehicle CTL group.

**Table 2:** Bone marrow micronucleus test results of Deg-AZM.

Time	Group	OPEM	OMP	NM Rate (‰)	PCE/(NCE + PCE)
24 h	vehicle CTL	20,000	4 ± 2	0.90 ± 0.58	0.71 ± 0.03
	120 mg/kg	20,000	4 ± 3	0.95 ± 0.72	0.67 ± 0.03
	300 mg/kg	20,000	3 ± 3	0.85 ± 0.70	0.71 ± 0.05
	750 mg/kg	20,000	5 ± 2	1.25 ± 0.40	0.69 ± 0.03
	positive CTL	20,000	60 ± 10 **	15.00 ± 2.59 **	0.54 ± 0.03 **
48 h	vehicle CTL	20,000	3 ± 2	0.80 ± 0.48	0.70 ± 0.03
	120 mg/kg	20,000	4 ± 3	0.90 ± 0.63	0.73 ± 0.03
	300 mg/kg	20,000	3 ± 2	0.65 ± 0.38	0.71 ± 0.06
	750 mg/kg	20,000	4 ± 2	1.00 ± 0.40	0.71 ± 0.04
	positive CTL	20,000	52 ± 12 **	13.05 ± 2.88 **	0.56 ± 0.04 **

Data are expressed as mean ± SD (n = 3). ** *p* < 0.01, vs. vehicle CTL group.

**Table 3:** The effect of Deg-AZM on chromosomal aberrations in CHL cells.

	Time	Group (µg/mL)	Types of Chromosomal Aberrations									TNAC	Aberration	DDR
			MG	MB	MD	G	B	Rc	R	Dic	Polyploid			
*+S9*	4 h	54.25	20	1	0	9	0	0	0	0	3	1	0.3	-
		108.5	14	0	0	17	0	0	0	1	4	1	0.3	-
		217	24	0	0	5	0	0	0	0	0	0	0.0	-
		434	48	2	0	11	0	0	0	0	2	2	0.7	-
		vehicle CTL	28	2	0	11	0	0	0	1	2	3	1.0	-
		cyclophosphamide	17	24	0	12	10	0	0	1	2	35	11.7 **	+
−*S9*	4 h	54.25	15	1	0	7	0	0	0	0	0	1	0.3	-
		108.5	9	0	0	12	0	0	0	1	3	0	0.0	-
		217	15	0	0	8	0	0	0	0	0	1	0.3	-
		434	14	0	0	12	0	0	0	0	0	0	0.0	-
		vehicle CTL	14	0	0	5	0	0	0	0	0	0	0.0	-
		mitomycin C	28	32	1	15	6	1	0	0	3	40	13.3 **	+
	24 h	54.25	7	1	0	6	0	0	0	0	0	1	0.3	-
		108.5	16	1	0	18	0	0	0	0	0	1	0.3	-
		217	11	1	0	8	0	0	0	0	0	1	0.3	-
		434	19	1	0	8	0	0	1	0	0	3	1.0	-
		vehicle CTL	16	1	0	15	0	0	1	0	0	2	0.7	-
		mitomycin C	25	25	1	19	18	0	0	0	0	43	14.3 **	+
	48 h	54.25	16	0	0	8	0	0	1	0	0	1	0.3	-
		108.5	18	0	0	13	0	0	0	0	4	0	0.0	-
		217	19	2	0	10	0	0	0	0	1	2	0.7	-
		434	24	1	0	14	0	0	0	0	0	1	0.3	-
		vehicle CTL	22	1	0	10	0	1	0	0	1	2	0.7	-
		mitomycin C	23	21	0	13	11	0	0	5	1	37	12.3 **	+

** *p* < 0.01, vs. vehicle CTL group. MG: Chromatid gap; MB: Chromatid break; MD: Chromatid deletion; G: Chromosome gap; B: Chromosome break; Rc: Chromatid rearrangement; R: Chromosome rearrangement; Dic: Dicentric chromosome; Polyploid: Polyploidy; TNAC: Total number of aberrant cells; DDR: Dose-dependent response (+: present, -: absent).

**Table 4:** Hematological and coagulation parameters in rats receiving 28-day repeated oral doses of Deg-AZM.

Parameters	Male Rats						Female Rats					
	CTL	10 mg/kg	25 mg/kg	50 mg/kg	150 mg/kg	600 mg/kg	CTL	10 mg/kg	25 mg/kg	50 mg/kg	150 mg/kg	600 mg/kg
WBC (×10^9^/L)	7.9 ± 1.5	6.2 ± 1.9	7.1 ± 1.5	7.6 ± 2.1	7.3 ± 1.7	8.5 ± 2.1	6 ± 1.6	4.8 ± 1.7	4.7 ± 1.8	6.7 ± 1.5	6.5 ± 1.4	6.1 ± 1.6
NEU (%)	1.3 ± 0.4	1 ± 0.2	1.2 ± 0.5	1.1 ± 0.4	1.2 ± 1	1.5 ± 1.2	0.7 ± 0.3	0.9 ± 0.3	1 ± 0.3	0.9 ± 0.3	0.7 ± 0.1	0.9 ± 0.3
NEU% (%)	16.6 ± 3.4	17.6 ± 5.5	16.6 ± 4.6	14.3 ± 3.9	15 ± 9.6	17 ± 8.5	12.9 ± 5.9	18.2 ± 3.9	23 ± 4.9	13.8 ± 5.5	11.5 ± 2.4	15.3 ± 4.2
LYM (×10^9^/L)	6.2 ± 1.4	4.9 ± 1.7	5.6 ± 1.2	6.2 ± 1.9	5.8 ± 1.4	6.7 ± 1.4	5 ± 1.5	3.8 ± 1.5	3.5 ± 1.5	5.5 ± 1.4	5.5 ± 1.4	4.9 ± 1.4
LYM% (%)	77.9 ± 4.5	78.2 ± 5.7	78.9 ± 4.8	81.9 ± 4.3	80.9 ± 10.2	79.7 ± 9.2	83.1 ± 6	78 ± 4	73.1 ± 4.2	82 ± 5.9	84.8 ± 2.8	80.4 ± 4.4
MONO (×10^9^/L)	0.2 ± 0	0.1 ± 0 *	0.2 ± 0.1	0.1 ± 0	0.1 ± 0.1	0.1 ± 0.1	0.1 ± 0	0.1 ± 0.1	0.1 ± 0	0.1 ± 0	0.1 ± 0	0.1 ± 0
MONO% (%)	2.6 ± 0.7	1.9 ± 0.6	2.1 ± 0.7	1.6 ± 0.4	1.7 ± 0.9	1.5 ± 0.6	1.8 ± 0.6	1.7 ± 0.5	1.3 ± 0.3	1.8 ± 0.4	1.7 ± 0.4	1.8 ± 0.5
EOS(×10^9^/L)	0.1 ± 0	0.1 ± 0	0.1 ± 0	0.1 ± 0 *	0.1 ± 0 **	0.1 ± 0 **	0.1 ± 0	0 ± 0	0 ± 0	0.1 ± 0	0.1 ± 0	0.1 ± 0
EOS% (%)	0.7 ± 0.2	0.8 ± 0.5	1 ± 0.6	1 ± 0.3	1 ± 0.3	0.8 ± 0.3	1.2 ± 0.3	0.7 ± 0.2	0.8 ± 0.3	1.1 ± 0.5	1 ± 0.3	1.3 ± 0.4
BASO (×10^9^/L)	0 ± 0	0 ± 0	0 ± 0	0 ± 0	0 ± 0	0 ± 0	0 ± 0	0 ± 0	0 ± 0	0 ± 0	0 ± 0	0 ± 0
BASO% (%)	0.2 ± 0.1	0.2 ± 0.1	0.2 ± 0.1	0.4 ± 0.1	0.3 ± 0.1	0.3 ± 0.1	0.3 ± 0.1	0.2 ± 0.1	0.1 ± 0.1	0.3 ± 0.1	0.3 ± 0.1	0.3 ± 0.1
RBC (×10^12^/L)	7.8 ± 0.4	7.5 ± 0.4	7.4 ± 0.6	7.8 ± 0.3	7.6 ± 0.3	7.8 ± 0.2	7.3 ± 0.2	7.4 ± 0.2	7.1 ± 0.6	7.3 ± 0.2	7.4 ± 0.3	7.2 ± 0.3
HGB (g/dL)	14.8 ± 0.6	14.5 ± 0.7	14.1 ± 1	15 ± 0.6	15.1 ± 0.7	15.2 ± 0.5	14.4 ± 0.5	13.7 ± 0.4	13.5 ± 0.4	14.3 ± 0.4	14.3 ± 0.6	14.2 ± 0.6
HCT% (%)	45.2 ± 1.8	44.4 ± 1.7	43.3 ± 3.2	44.2 ± 1.7	44.3 ± 1.7	44.1 ± 1.1	41 ± 1.4	41.3 ± 1.3	40.2 ± 3.4	40.7 ± 1.4	40.9 ± 1.7	40.5 ± 1.7
MCV (fL)	52 ± 2.2	59 ± 2	58.5 ± 1.9	56.8 ± 1.4	58.3 ± 1.5 **	56.7 ± 1	56.2 ± 1.3	56.1 ± 1.7	56.4 ± 1.5	56 ± 1.4	55.5 ± 1.3	56 ± 2.2
MCH (pg)	18.9 ± 0.9	19.2 ± 0.4	19 ± 0.6	19.3 ± 0.7	19.9 ± 0.7	19.5 ± 0.4	19.7 ± 0.5	18.5 ± 0.6	19 ± 1.5	19.7 ± 0.5	19.4 ± 0.5	19.6 ± 0.7
MCHC (g/dL)	32.6 ± 0.3	32.6 ± 0.7	32.6 ± 0.7	34 ± 0.6	34.1 ± 0.6	34.3 ± 0.6	35.1 ± 0.4	33 ± 0.3	33.7 ± 2.8	35.1 ± 0.3	35 ± 0.7	35 ± 0.4
RDW% (%)	12.5 ± 0.5	12.4 ± 0.4	12.7 ± 0.7	12 ± 0.3	11.7 ± 0.3	11.9 ± 0.6	11.4 ± 0.6	12.1 ± 0.4	12.5 ± 0.4	11.2 ± 0.5	11.6 ± 0.5	11.8 ± 0.4
PLT (×10^9^/L)	1198 ± 99	1073 ± 173	1117 ± 145	1158 ± 89	1139 ± 69 *	1155 ± 74 *	1155 ± 111	1079 ± 87	1075 ± 181	1131 ± 103	1126 ± 122	1127 ± 63
PDW% (%)	42.7 ± 1.9	43.1 ± 1.5	42.9 ± 1.9	47 ± 2.3	47.6 ± 1.1	47.2 ± 1.8	47.1 ± 1.1	43 ± 1.3	43.7 ± 1.6	47.4 ± 2.1	46.7 ± 1.5	46.7 ± 0.9
RETIC% (%)	3.7 ± 0.5	3.9 ± 0.6	4.4 ± 0.8	3.4 ± 0.4	3.6 ± 0.5	3.4 ± 0.7	3.2 ± 0.8	3.4 ± 0.4	4.1 ± 1.2	3.2 ± 0.8	3.6 ± 0.8	3.6 ± 0.6
PT (s)	10.6 ± 0.4	10.6 ± 0.6	10.5 ± 0.5	12.4 ± 0.9	11.7 ± 0.5	11.6 ± 1	10.7 ± 0.4	10.7 ± 0.6	10.7 ± 0.4	11.1 ± 0.9	11.2 ± 0.7	10.8 ± 0.5
APTT (s)	11.7 ± 1.4	11.9 ± 2.3	11.3 ± 1.4	16.1 ± 0.7	15.4 ± 1.7	15.5 ± 1.1	14.1 ± 2	13 ± 1.6	12.9 ± 1.4	14 ± 1.6	12.9 ± 1.5	14 ± 1.5
FIB (mg/dL)	152 ± 6	152 ± 8	161 ± 13	148 ± 5	154 ± 8	155 ± 11	134 ± 12	131 ± 10	134 ± 11	135 ± 7	137 ± 9	139 ± 7
TT (s)	40.4 ± 6	38.6 ± 6.7	39.4 ± 7.2	34.9 ± 1.8	34.4 ± 1.5	34.3 ± 1.6	34.7 ± 4.9	36.3 ± 4	35.9 ± 3.6	35.9 ± 2.4	34.1 ± 2.8	35.7 ± 3.6

Data are presented as mean ± SD (n = 4 animals/group). One-way ANOVA with Dunnett’s post hoc test was used to compare all groups against the CTL (control) group. * *p* < 0.05, ** *p* < 0.01 vs. CTL. Abbreviations: WBC, white blood cell count; NEU, neutrophil count; NEU%, neutrophil percentage; LYM, lymphocyte count; LYM%, lymphocyte percentage; MONO, monocyte count; MONO%, monocyte percentage; EOS, eosinophil count; EOS%, eosinophil percentage; BASO, basophil count; BASO%, basophil percentage; RBC, red blood cell count; HGB, hemoglobin concentration; HCT, hematocrit; MCV, mean corpuscular volume; MCH, mean corpuscular hemoglobin; MCHC, mean corpuscular hemoglobin concentration; RDW, red cell distribution width; PLT, platelet count; PDW, platelet distribution width; RETIC, reticulocyte percentage; PT, prothrombin time; APTT, activated partial thromboplastin time; FIB, fibrinogen concentration; TT, thrombin time.

**Table 5:** The serum biochemical parameters in rats receiving 28-day repeated oral doses of Deg-AZM.

Parameters	Male Rats						Female Rats					
	CTL	10 mg/kg	25 mg/kg	50 mg/kg	150 mg/kg	600 mg/kg	CTL	10 mg/kg	25 mg/kg	50 mg/kg	150 mg/kg	600 mg/kg
AST (U/L)	97 ± 19	96 ± 13	88 ± 8	98 ± 8	107 ± 21	100 ± 18	100 ± 16	114 ± 50	102 ± 27	101 ± 15	100 ± 11	87 ± 11
ALT (U/L)	35 ± 7	35 ± 10	33 ± 7	39 ± 9	43 ± 7	42 ± 9	33 ± 6	33 ± 15	29 ± 11	37 ± 11	34 ± 4 *	31 ± 9 **
ALP (U/L)	199 ± 27	197 ± 29	164 ± 27 *	175 ± 43	167 ± 27	192 ± 31	91 ± 22	79 ± 15	105 ± 21	75 ± 24	74 ± 23	77 ± 24
TBIL (µmol/L)	2.6 ± 0.5	2.4 ± 0.5	2.4 ± 0.4	2.6 ± 0.7	2.6 ± 0.6	2.5 ± 0.8	3.1 ± 0.6	3.1 ± 0.6	2.5 ± 0.6	3.2 ± 0.8	2.9 ± 0.6	3 ± 0.9
TP (g/L)	61.6 ± 1.4	62 ± 2.5	61.4 ± 3.1	61.3 ± 1.9	62.6 ± 2.5	63.3 ± 2.4	70.8 ± 4	71.8 ± 3.9	72.2 ± 3.2	69.1 ± 5.1	69.5 ± 3.9	68.4 ± 3.6
ALB (g/L)	31.1 ± 1.4	31.4 ± 0.9	31.3 ± 1.7	28 ± 1.6	28.6 ± 1.1	28.2 ± 0.8	37.6 ± 2.5	39.6 ± 3.5	38.7 ± 2.6	32.8 ± 2.7	33.9 ± 2.1	32.9 ± 2.2
GLO (g/L)	30.5 ± 1.4	30.6 ± 2.4	30.1 ± 1.8	33.3 ± 0.7	34.1 ± 1.7	35.1 ± 1.9	33.2 ± 1.7	32.2 ± 1.5	33.5 ± 1.7	36.3 ± 2.7	35.6 ± 2.3	35.5 ± 2.3
A/G	1 ± 0.1	1 ± 0.1	1 ± 0.1	0.8 ± 0.1	0.9 ± 0.1	0.8 ± 0	1.1 ± 0.1	1.2 ± 0.1	1.2 ± 0.1	0.9 ± 0	1 ± 0.1	0.9 ± 0.1
BUN (mmol/L)	5.6 ± 1	5.4 ± 0.5	5.9 ± 1.7	4.6 ± 0.9	4.3 ± 0.4	4.2 ± 0.7	6.2 ± 0.5	5.8 ± 1.2	6 ± 0.9	5.7 ± 0.7	5.8 ± 1.1	5.3 ± 0.4
CREA (µmol/L)	40 ± 3	45 ± 4 *	43 ± 4	39 ± 7	36 ± 4	39 ± 9	60 ± 8	57 ± 8	60 ± 14	50 ± 5	53 ± 17	51 ± 9
CHOL (mmol/L)	1.8 ± 0.1	1.8 ± 0.2	2 ± 0.5	1.5 ± 0.5	1.8 ± 0.4	1.9 ± 0.4	1.9 ± 0.4	1.9 ± 0.3	2 ± 0.4	1.8 ± 0.5	1.9 ± 0.5	2.1 ± 0.4
TGL (mmol/L)	0.7 ± 0.2	0.6 ± 0.2	0.8 ± 0.6	0.4 ± 0.2	0.4 ± 0.2	0.4 ± 0.2	0.5 ± 0.1	0.5 ± 0.1	0.4 ± 0.1	0.4 ± 0.1	0.4 ± 0.2	0.4 ± 0.1
GLU (mmol/L)	6.3 ± 0.5	6.8 ± 0.3 *	7 ± 0.3 **	6.2 ± 0.5	6 ± 0.7	6.4 ± 0.8	7.5 ± 0.7	7.3 ± 0.9	7.2 ± 0.5	5.6 ± 0.6	5.5 ± 0.6	6.5 ± 0.6
CK (U/L)	322 ± 58	316 ± 89	314 ± 48	301 ± 117	314 ± 89	261 ± 133	312 ± 172	253 ± 102	311 ± 175	201 ± 76	359 ± 260	184 ± 65
K (mmol/L)	4.6 ± 0.2	4.6 ± 0.2	4.7 ± 0.2	4.5 ± 0.3	4.7 ± 0.3	4.6 ± 0.4	4.3 ± 0.2	4.2 ± 0.3	4.4 ± 0.4	4.2 ± 0.3	4.4 ± 0.3	4.2 ± 0.3
NA (mmol/L)	147 ± 2	146 ± 3	147 ± 2	149 ± 1	149 ± 1	149 ± 2	147 ± 2	147 ± 2	148 ± 2	147 ± 1	147 ± 2	147 ± 2
CL (mmol/L)	105 ± 2	106 ± 2	106 ± 1	108 ± 1	108 ± 1	107 ± 1	106 ± 1	107 ± 1	107 ± 2	107 ± 1	106 ± 1	107 ± 2
GGT (U/L)	2.9 ± 1.1	2.6 ± 1	2.6 ± 1.1	2.4 ± 1.7	1.7 ± 1.3	1.4 ± 1.4	2.4 ± 0.6	2.8 ± 0.6	2.9 ± 0.9	1.4 ± 1.3	1.7 ± 1.3	1.9 ± 2
CA (mmol/L)	2.6 ± 0.1	2.6 ± 0.1	2.6 ± 0.1	2.5 ± 0.1	2.5 ± 0.1	2.6 ± 0.1	2.7 ± 0.1	2.7 ± 0.1	2.7 ± 0.1	2.6 ± 0.1	2.6 ± 0.1	2.6 ± 0.1
PHOS (mmol/L)	3 ± 0.2	3 ± 0.2	3 ± 0.2	2.7 ± 0.2	2.8 ± 0.1	2.7 ± 0.2	2.6 ± 0.2	2.7 ± 0.2	2.8 ± 0.4	2.2 ± 0.1	2.4 ± 0.3	2.2 ± 0.2

Data are expressed as mean ± SD (n = 20 animals/group). One-way ANOVA with Dunnett’s post hoc test was used to compare all groups against the CTL group. * *p* < 0.05, ** *p* < 0.01, vs. CTL group. Abbreviations: AST, aspartate aminotransferase; ALT, alanine aminotransferase; ALP, alkaline phosphatase; TBIL, total bilirubin; TP, total protein; ALB, albumin; GLO, globulin; A/G, albumin-to-globulin ratio; BUN, blood urea nitrogen; CREA, creatinine; CHOL, total cholesterol; TGL, triglycerides; GLU, glucose; CK, creatine kinase; K, potassium; NA, sodium; CL, chloride; GGT, gamma-glutamyl transferase; CA, calcium; PHOS, phosphorus.

**Table 6:** Pharmacokinetic parameters of Deg-AZM in plasma of rats following 28-day repeated oral administration at different doses. Data are expressed as means ± SD (n = 16).

Groups	Parameters	10 mg/kg		25 mg/kg		50 mg/kg		150 mg/kg		600 mg/kg	
		Day1	Day28	Day1	Day28	Day1	Day28	Day1	Day28	Day1	Day28
Male	AUC_0–t_ (μg/mL·h)	0.749 ± 0.18	1.27 ± 0.23	3.54 ± 0.98	5.20 ± 0.85	8.13 ± 1.72	13.10 ± 2.73	37.97 ± 3.41	31.40 ± 4.58	258.04 ± 58.48	200.7 ± 35.32
	T_1/2_ (h)	7.48 ± 4.48	1.94 ± 0.73	2.68 ± 1.43	1.25 ± 0.31	1.49 ± 0.44	1.5 ± 0.29	2.53 ± 0.42	3.45 ± 1.80	4.97 ± 3.76	3.57 ± 1.09
	T_max_ (h)	2.38 ± 2.50	1.75 ± 0.50	1.00 ± 0.707	1.25 ± 0.87	0.75 ± 0.29	0.75 ± 0.29	1.13 ± 0.63	1.38 ± 0.75	1.25 ± 0.50	1.25 ± 0.87
	C_max_ (μg /mL)	0.22 ± 0.092	0.51 ± 0.033	0.94 ± 0.30	2.25 ± 1.33	2.79 ± 0.42	2.87 ± 1.65	8.07 ± 0.85	5.74 ± 0.94	27.01 ± 3.52	22 ± 2.82
Female	AUC_0–t_ (μg/mL·h)	1.67 ± 0.55	1.15 ± 0.31	5.11 ± 0.83	3.20 ± 0.30	11.15 ± 2.78	9.35 ± 2.33	54.19 ± 11.83	22.44 ± 6.59	89.66 ± 35.01	145 ± 44.0
	T_1/2_ (h)	1.54 ± 0.06	1.71 ± 0.88	1.72 ± 0.46	1.00 ± 0.23	1.67 ± 0.11	1.31 ± 0.23	2.05 ± 0.90	3.53 ± 1.79	4.35 ± 1.65	3.58 ±0.60
	T_max_ (h)	0.63 ± 0.25	1.17 ± 0.97	1.00 ± 0.71	0.50	0.42 ± 0.17	0.50 ± 0.00	0.71 ± 0.88	0.42 ± 0.17	0.33 ± 0.19	0.33 ± 0.19
	C_max_ (μg /mL)	0.67 ± 0.22	0.64 ± 0.25	1.69 ± 0.21	1.80 ± 0.31	3.51 ± 2.11	2.90 ± 0.93	14.09 ± 2.87	4.00 ± 1.10	14.54 ± 4.95	20.71 ± 4.36

**Table 7:** The organ-to-body weight ratio in rats receiving 28-day repeated oral doses of Deg-AZM.

Group		Heart	Liver	Spleen	Lung	Kidney	Stomach	Adrenal Gland	Thymus	Brain	Testicle	Epididymis	Oophoron	Uterus
CTL	♂	0.40 ± 0.04	2.85 ± 0.20	0.20 ± 0.02	0.40 ± 0.06	0.68 ± 0.03	0.48 ± 0.05	0.01 ± 0.00	0.13 ± 0.03	0.51 ± 0.03	0.81 ± 0.06	0.24 ± 0.03	—	—
	♀	0.38 ± 0.03	2.79 ± 0.18	0.19 ± 0.03	0.49 ± 0.04	0.64 ± 0.04	0.59 ± 0.04	0.06 ± 0.11	0.16 ± 0.03	0.80 ± 0.1	—	—	0.04 ± 0.01	0.19 ± 0.03
10 mg/kg	♂	0.37 ± 0.04	2.83 ± 0.11	0.19 ± 0.02	0.37 ± 0.02	0.61 ± 0.1 *	0.47 ± 0.04	0.01 ± 0.00	0.13 ± 0.03	0.49 ± 0.05	0.8 ± 0.09	0.24 ± 0.02	—	—
	♀	0.41 ± 0.04	2.89 ± 0.19	0.21 ± 0.02	0.48 ± 0.05	0.64 ± 0.05	0.58 ± 0.07	0.03 ± 0.00	0.15 ± 0.02	0.8 ± 0.07	—	—	0.03 ± 0.01	0.23 ± 0.07
25 mg/kg	♂	0.36 ± 0.05	2.98 ± 0.26	0.19 ± 0.02	0.38 ± 0.04	0.72 ± 0.18	0.49 ± 0.05	0.01 ± 0.00	0.13 ± 0.01	0.49 ± 0.04	0.72 ± 0.17	0.22 ± 0.04	—	—
	♀	0.39 ± 0.03	2.83 ± 0.2	0.19 ± 0.02	0.47 ± 0.03	0.65 ± 0.05	0.6 ± 0.03	0.03 ± 0.00	0.16 ± 0.03	0.78 ± 0.05	—	—	0.04 ± 0.01	0.24 ± 0.11
50 mg/kg	♂	0.40 ± 0.03	2.83 ± 0.18	0.21 ± 0.02	0.39 ± 0.02	0.72 ± 0.04	0.50 ± 0.03	0.02 ± 0.00	0.14 ± 0.03	0.53 ± 0.02	0.86 ± 0.07	0.27 ± 0.03	—	—
	♀	1.02 ± 0.08	7.20 ± 0.61	0.62 ± 0.08	1.19 ± 0.11	1.74 ± 0.13	1.53 ± 0.11	0.08 ± 0.01	0.50 ± 0.09	1.93 ± 0.03	—	—	0.05 ± 0.00	0.18 ± 0.04
150 mg/kg	♂	0.41 ± 0.03	3.04 ± 0.25	0.21 ± 0.03	0.38 ± 0.02	0.73 ± 0.04	0.56 ± 0.10	0.02 ± 0.00	0.14 ± 0.03	0.53 ± 0.03	0.89 ± 0.06	0.28 ± 0.02	—	—
	♀	1.05 ± 0.21	6.95 ± 0.72	0.56 ± 0.07	1.15 ± 0.15	1.74 ± 0.17	1.51 ± 0.11	0.08 ± 0.01	0.44 ± 0.17	1.95 ± 0.11	—	—	0.05 ± 0.01	0.21 ± 0.05
600 mg/kg	♂	0.40 ± 0.02	3.3 ± 0.21 **	0.20 ± 0.02	0.38 ± 0.02	0.73 ± 0.04	0.58 ± 0.03	0.02 ± 0.00	0.15 ± 0.03	0.54 ± 0.04	0.83 ± 0.22	0.28 ± 0.06	—	—
	♀	0.45 ± 0.12	3.24 ± 0.18 **	0.22 ± 0.03	0.45 ± 0.04	0.71 ± 0.03	0.69 ± 0.07	0.03 ± 0.00	0.18 ± 0.03	0.76 ± 0.04	—	—	0.04 ± 0.01	0.22 ± 0.04

Data are expressed as mean ± SD (n = 20). * *p* < 0.05, ** *p* < 0.01, vs. CTL group. “—” indicated not applicable.

**Table 8:** Pregnancy and delivery in pregnant rats after oral administration of Deg-AZM.

Parameter	Group			
	CTL	50 mg/kg	150 mg/kg	600 mg/kg
Pregnancy rate (%)	91.3 (21/23)	95.7 (22/23)	95.8 (23/24)	87.5 (21/24)
False pregnancy rate (%)	8.7 (2/23)	4.3 (1/23)	4.2 (1/24)	12.5 (3/24)
Premature birth rate (%)	0.0 (0/23)	0.0 (0/23)	0.0 (0/24)	0.0 (0/24)
Abortion rate (%)	0.0 (0/23)	0.0 (0/23)	0.0 (0/24)	0.0 (0/24)
Death rate (%)	0.0 (0/23)	0.0 (0/23)	0.0 (0/24)	0.0 (0/24)

**Table 9:** Pharmacokinetic parameters of Deg-AZM in the plasma of pregnant rats following oral administration. Data are expressed as the means ± SD (n = 6).

Parameters	50 mg/kg		150 mg/kg		600 mg/kg	
	GD6	GD15	GD6	GD15	GD6	GD15
AUC_0–t_ (μg/mL·h)	11.3 ± 6.1	6.0 ± 1.4	45.3 ± 11.3	24.9 ± 5.4	192.7 ± 4.7	110.3 ± 2.6
T_1/2_ (h)	2.4 ± 0.6	2.7 ± 1.4	4.0 ± 2.2	4.3 ± 2.8	3.5 ± 1.6	4.6 ± 2.3
T_max_ (h)	0.7 ± 0.3	1.0 ± 0.9	0.5 ± 0.0	1.0 ± 0.9	0.2 ± 0.2	0.5 ± 0.0
C_max_ (μg /mL)	2.8 ± 1.2	1.7 ± 0.3	9.6 ± 1.6	5.6 ± 3.2	22.4 ± 6.9	18.8 ± 4.9

## Data Availability

The data presented in this study are available on request from the corresponding author due to privacy and ethical reasons.

## Supplementary Materials

The following supporting information can be downloaded at: https://www.mdpi.com/article/10.3390/biomedicines13112600/s1, Table S1. Maternal Organ Weights, Reproductive Findings, and Fetal Morphology and Sex Ratio following single oral administration of Deg-AZM: title; Table S2. Skeletal survey Parameters in fetal rats following single oral administration of Deg-AZM; Table S3. Internal organ examination parameters in fetal rats following single oral administration of Deg-AZM.

## Abbreviations

The following abbreviations are used in this manuscript:

5-HT4	5-Hydroxytryptamine receptor subtype 4
+*S9*	with *S9* mix
−*S9*	without *S9* mix
A/G	albumin-to-globulin ratio
ALB	albumin
ALP	alkaline phosphatase
ALT	alanine aminotransferase
APTT	activated partial thromboplastin time
AST	aspartate aminotransferase
AUC	area under the concentration–time curve
BUN	blood urea nitrogen
CHL	Chinese hamster lung
CHOL	total cholesterol
CK	creatine kinase
C_max_	peak concentration
CMC-Na	Carboxymethylcellulose sodium
CPN	chronic progressive nephropathy
CREA	creatinine
CTL	control
Deg-AZM	Deglycosylated-azithromycin
DMSO	dimethyl sulfoxide
EFD	Embryo–fetal development
EOS	eosinophil
F-actin	filamentous actin
FIB	fibrinogen
G-actin	globular actin
GGT	gamma-glutamyl transferase
GD	gestation day
GLO	globulin
GLU	glucose
H&E	hematoxylin and eosin
HCT	hematocrit
HGB	hemoglobin concentration
IVC	inferior vena cava
LD_50_	median lethal dose
MCH	mean corpuscular hemoglobin
MCHC	mean corpuscular hemoglobin concentration
MCV	mean corpuscular volume
MN	micronuclei
NBF	neutral buffered formalin
NCE	normochromic erythrocytes
PCE	polychromatic erythrocytes
PDW	platelet distribution width
PLT	platelet count
PT	prothrombin time
RBC	red blood cell count
RDW	red cell distribution width
RETIC	reticulocyte
SD	Sprague-Dawley
SM22α	Transgelin
STC	Slow-transit constipation
T_1/2_	terminal elimination half-life
TBIL	total bilirubin
TGL	triglycerides
T_max_	time to maximum concentration
TP	total protein
TT	thrombin time
WBC	white blood cell
