# Supplementary material for: The Genetic and Embryo–Fetal Developmental Toxicity Profile of the Novel Transgelin Agonist Deg-AZM: Ames, Micronucleus, Chromosomal Aberration, and Rat EFD Studies
Source: Biomedicines. 2025 Oct 23;13(11):2600. doi: 10.3390/biomedicines13112600 (PMC12650075; doi:10.3390/biomedicines13112600)
Supplement: Supplementary file 1 [file biomedicines-13-02600-s001.zip › biomedicines-3918790-supplementary.pdf]

Table S1. Maternal Organ Weights, Reproductive Findings, and Fetal Morphology and Sex Ratio following single oral administration of Deg-AZM.

**Parameter**		**Group**			
		**CTL**	**50 mg/kg**	**150 mg/kg**	**600 mg/kg**
**Maternal Endpoints**					
Organ Wet Weight	Brain	1.90±0.11	1.90±0.09	1.93±0.12	1.90±0.13
	Ovary (R)	0.065±0.014	0.064±0.015	0.065±0.014	0.066±0.014
	Ovary (L)	0.063±0.015	0.062±0.012	0.061±0.0145	0.07±0.016
Number of Corpora Lutea		16.7±3.9	16.8±2.2	16.8±2.5	17.2±2.7
Number of Implantation Sites		14.8±4.2	14.4±3.2	13.7±3.2	14.8±2.7
Litter Weight (g)		81.4±23.4	76.2±17.0	72.1±20.9	79.2±16.6
Placental Weight (g)		0.5±0.1	0.5±0.1	0.6±0.2	0.5±0.1
**Fetal Development**					
Body Weight (g)	♂	3.7±0.3	3.7±0.3	3.6±0.3	3.7±0.3
	♀	3.5±0.4	3.5±0.3	3.4±0.4	3.5±0.2
Body Length (mm)	♂	34.5±1.3	35.2±1.6	34.6±1.4	34.8±0.8
	♀	33.7±1.5	34.3±1.3	33.5±2.0	33.9±0.8
Sex Ratio (♂:♀)		1.0 (152:155)	0.8 (131:171)	0.9 (144:155)	0.9 (147:156)

Data were expressed as the means ± SD.

Table S2. Skeletal survey Parameters in fetal rats following single oral administration of Deg-AZM.

Parameter	Group	
	CTL	600 mg/kg
**Limb Ossification (Count)**		
Metacarpals	7.0±0.7	6.9±0.7
Metatarsals	8.0±0.0	8.0±0.1
Sternum	5.7±0.2	5.6±0.4
Sacral & Caudal, Total	7.5±0.4	7.3±0.6
**Craniofacial Ossification (%, litter)**		
Parietal Bones, Incomplete	1.4±4.8 (2/21)	4.0±8.4 (5/21)
Interparietal Bone, Incomplete	11.3±19.3 (7/21)	6.8±10.9 (7/21)
Occipital Bone, Incomplete	3.7±7.1 (5/21)	4.3±7.4 (6/21)
Hyoid Bone, Unossified	23.6±20.7 (15/21)	16.6±18.6 (13/21)
Hyoid Bone, Incomplete	7.1±10.0 (9/21)	2.9±6.4 (4/21)
**Vertebral Centra & Arch Ossification (%, litter)**		
Thoracic Centrum, Dumbbell	1.9±6.6 (2/21)	2.1±6.7 (2/21)
Thoracic Centrum, Split	0.0±0.0 (0/21)	1.3±4.0 (2/21)
Sacral Arch, Incomplete	31.3±29.9 (14/21)	31.0±27.6 (15/21)
**Sternebrae & Ribs (%, litter)**		
Ossification, Incomplete	55.5±23.9 (20/21)	57.3±18.1 (21/21)
Ossification, Dumbbell	12.8±23.9 (10/21)	14.7±17.4 (12/21)
Ossification, Asymmetric	0.6±2.7 (1/21)	2.1±5.6 (3/21)
Ossification, Split	2.7±7.9 (3/21)	0.0±0.0 (0/21)
Lumbar Rib	0.5±2.2 (1/21)	0.0±0.0 (0/21)
Skeletal Variation	83.1±18.1 (21/21)	79.9±18.6 (21/21)
Skeletal Malformation	0.0±0.0 (0/21)	0.0±0.0 (0/21)

Data were expressed as the means ± SD.

Table S3. Internal organ examination parameters in fetal rats following single oral administration of Deg-AZM.

Parameter	Group	
	CTL	600 mg/kg
Transposition of Umbilical Arteries (%, litter)	0.6±2.8 (1/20)	1.3±4.0 (2/21)
Thymic Remnant (%, litter)	4.5±8.9 (5/20)	6.3±13.4 (5/21)
Renal Pyelectasis, Right (%, litter)	9.7±11.4 (10/20)	6.4±8.7 (8/21)
Renal Pyelectasis, Left (%, litter)	8.9±14.2 (7/20)	9.8±12.4 (11/21)
Dilated Ureter (%, litter)	0.7±3.2 (1/20)	0.7±3.1 (1/21)
Visceral Variation (%, litter)	18.6±16.7 (14/20)	20.3±19.4 (17/21)
Visceral Malformation (%, litter)	0.0±0.0 (0/20)	0.0±0.0 (0/21)

Data were expressed as the means ± SD.
